# Pilot Investigation of PTSD, Autonomic Reactivity, and Cardiovascular Health in Physically Healthy Combat Veterans

**DOI:** 10.1371/journal.pone.0162547

**Published:** 2016-09-08

**Authors:** Ashley N. Clausen, Robin L. Aupperle, Jason-Flor V. Sisante, David R. Wilson, Sandra A. Billinger

**Affiliations:** 1 Department of Psychology, University of Tulsa, Tulsa, OK, United States of America; 2 Laureate Institute for Brain Research, Tulsa, OK, United States of America; 3 University of Kansas Medical Center, Department of Physical Therapy and Rehabilitation Science, Kansas City, KS, United States of America; Harvard Medical School, UNITED STATES

## Abstract

Posttraumatic stress disorder (PTSD), and combat-related PTSD in particular, has been associated with increased rates of cardiovascular disease, and cardiovascular-related death. However, less research has examined possible factors that may link PTSD to poorer cardiovascular health in combat veteran populations. The current pilot study investigated whether psychological symptomology and autonomic reactivity to emotional scripts would relate to poorer cardiovascular health in combat veterans without a current diagnosis of cardiovascular disease. Male veterans (N = 24), who served in combat since Operation Iraqi Freedom, completed a semi-structured interview and self-report measures to assess psychological symptomology. Autonomic reactivity, measured using heart rate variability (HRV; low to high frequency ratio), was obtained during script-driven imagery of emotional memories. Cardiovascular health was assessed using flow-mediated dilation (FMD) of the brachial artery. Correlational analyses and discriminant analysis were used to assess the relationship between psychological symptoms (PTSD, depression, anger, as measured via self-report), autonomic reactivity to emotional scripts (HRV), and FMD. Overall, veterans in the current study showed poor cardiovascular health despite their relatively young age and lack of behavioral risk factors, with 15/24 exhibiting impaired FMD (FMD < 5%). Psychological symptomology was not associated with FMD; whereas autonomic reactivity to emotional (compared to neutral) scripts was found to relate to FMD. Autonomic reactivity to negative scripts correctly classified 76.5% of veterans as having impaired versus normative FMD. Results from this pilot study highlight the importance of cardiovascular screening with combat veterans despite psychological diagnosis. Results also support the need for longitudinal research assessing the use of autonomic reactivity to emotionally valenced stimuli as a potential risk factor for poorer cardiovascular health.

## Introduction

It is estimated that approximately 90 percent of adults within the United States will experience a traumatic event [1]. Of those, only an estimated eight percent will go on to develop PTSD [2]. However, when compared to the general adult population, veterans who experience combat are over three times more likely to develop PTSD [3, 4], with prevalence estimates of 20–30 percent [3–5]. Due to the recent conflicts in Iraq and Afghanistan, the prevalence of PTSD for combat-exposed veterans in the United States has doubled from 1997–2005, and continues to rise [6], highlighting the importance of PTSD research in veteran populations. PTSD is characterized by symptoms of re-experiencing, avoidance, as well as hyperarousal and hypervigilance. The latter symptoms involve increased and prolonged emotional and autonomic reactivity (including cardiovascular response) to trauma-related cues [7–9]. There is evidence that prolonged autonomic reactivity may be associated with increased prevalence of physical health problems such as musculoskeletal disorders, and hypertension [8, 9].

Previous research indicates that combat exposure [10, 11] and PTSD symptoms [12–20], as well as comorbid disorders such as depression [21, 22] are associated with a higher risk for developing cardiovascular (CV) disease. Retrospective and cross-sectional research has identified a relationship between combat-related PTSD and CV risk factors including hypertension, and dyslipidemia [23, 24], as well as a diagnosis of CV disease and CV-related death [12, 14–16]. Notably, these relationships remain significant when controlling for potential lifestyle factors including smoking status and substance use [13, 17, 25, 26], and other psychological symptoms, such as depression [13, 25]. Thus, similar to findings concerning anxious symptoms and CV health in non-PTSD populations [26], this literature suggests that mechanisms other than conventional risk factors may be responsible for the relationship between PTSD and CV disease. The lifetime prevalence rate for CV disease in the United States is approximately 33 percent [27]. Given the high prevalence rate, it is important to gain a more clear understanding of the factors associated with the development of CV disease.

Most studies investigating the relationship between combat-related PTSD, and CV health have been epidemiological, investigating rates of diagnosed CV disease for those with and without combat exposure and PTSD. The studies that include assessment of CV health have most often relied on resting blood pressure, heart rate (HR), or lipid levels. Flow mediated dilation (FMD) provides unique and potentially predictive information concerning CV health. Specifically, FMD uses Doppler ultrasound to measure the vasodilatory response of an artery after an increase in luminal blood flow (flow within the artery), and is an assessment of endothelial function [28]. FMD is calculated as the peak arterial diameter from the baseline value (percent dilation) when exposed to increased blood flow [29]. Endothelial dysfunction, as measured by FMD, is one indicator of the development of atherosclerotic vascular disease [30]. FMD has been validated with angiography diagnosis of CV disease [31], and has been shown to be predictive of CV events, even after controlling for conventional risk factors [32]. Prior research utilizing FMD suggests that FMD dilation less than five percent is classified as impaired FMD, and is associated with an increased risk for the development of CV disease, whereas FMD dilation of individuals without CV disease is approximately seven percent [33]. In addition, FMD has been used to assess CV health in atherosclerotic vascular disease [34], anxiety disorders [35–38], and PTSD within police personnel [39]. While the summation of the current literature suggests a significant relationship between PTSD and CV health, research has yet to explore CV health via FMD in combat veteran populations. Furthermore, potential mechanisms for the development of CV disease in individuals with PTSD remains unclear [40].

Prior research suggests several possible links between PTSD and poorer CV health including dysreguation of the hypothalamic-pituitary-adrenal axis, which impacts corticosteroid release (which in turn influences the immune system, and lipid and glucose metabolism) and autonomic nervous system regulation (which in turn influences CV responses via regulation of heart rate, blood pressure, respiration, etc. [41]). Dysregulation of the vagus nerves, closely associated with autonomic function, has been shown to precede the development of CV disease [27, 42–45], making autonomic regulation a prime target for study. Prior research has established that increased sympathetic reactivity to emotional provocation (hyperarousal) is associated with combat-related PTSD and is considered a hallmark symptom of PTSD [16, 46–52]. Laboratory studies have objectively quantified this hyperarousal during script-driven imagery, a form of emotional provocation, using measures of sympathetic arousal [46, 48–52]. However, measurement of only sympathetic activity limits generalization of findings regarding autonomic reactivity. Heart rate variability (HRV) provides a measure of parasympathetic activity or the sympathetic/parasympathetic balance. Previous studies have reported combat-related PTSD to relate to depressed baseline heart rate variability (HRV) compared to those without PTSD [53–56]. However, it has yet to be determined whether autonomic reactivity relates to specific indicators of CV health, such as FMD, in veterans with PTSD.

The current pilot study aimed to explore how psychological symptoms (PTSD, depression), autonomic reactivity (HRV) to emotional events, and combat-exposure may relate to FMD in a veteran sample. A focus on relatively young, and physically healthy combat veterans was to allow for examination of FMD prior to the development of CV disease or other chronic health problems. It was hypothesized that greater PTSD symptoms would relate to more impaired FMD. Further, we hypothesized that increased autonomic reactivity during emotional provocation (specifically, script driven imagery of combat events) would significantly predict impaired FMD (FMD < 5% [33]), above and beyond that of psychological symptoms and combat exposure.

## Materials and Methods

### Participants

Participants included male combat veterans (N = 24; mean age = 32.75, SD = 7.69) who served since the onset of Operation Iraqi Freedom (OIF), with varying levels of PTSD symptomatology (7 diagnosed with full PTSD, 11 with partial PTSD; 6 not meeting PTSD diagnosis; definition of partial PTSD provided below). Exclusion criteria included active suicidal plan or intent, substance abuse within the past six months, schizophrenia or bipolar I disorder (confirmed via the Mini International Neuropsychiatric Inventory [57]), history of moderate to severe head injury (loss of consciousness >30 minutes or post-traumatic amnesia >1day), neurological disorder, CV disease, any medical condition directly affecting CV health, or use of medications within 30 days known to affect CV health. In addition, no participants included in the current study were taking psychotropic medication. Exclusion criteria for the current study were meant to limit possible confounding variables that may influence emotional processing and/or CV health, but to not exclude some of the more common comorbidities associated with PTSD (e.g., mild traumatic brain injury [mTBI], depression, other anxiety disorders).

Participants were recruited via advertisements in the general community (i.e., radio, newspaper, and Facebook) and on local college campuses (i.e., via emails, flyers, etc.), and by providing informational flyers to clinicians at local VA hospitals. This study was approved by the University of Kansas Medical Center and the University of Missouri—Kansas City Institutional Review Boards. All participants provided written, signed consent.

### Procedures

The current study procedures described below were conducted across two study visits. During the first visit, participants completed a clinical assessment to assess symptoms of PTSD (described below). Participants were asked to return for a second study visit within six months (ranging from one week to six months) to complete the remainder of the clinical assessment, assessment of CV health, and the script-driven imagery task (described below). During the second study visit, participants completed the assessments in the following order: assessment of CV health, script-driven imagery task, and clinical assessment (i.e., self-reported questionnaires). Assessment of CV health was always conducted prior to script-driven imagery to minimize subject burden due to fasting, as well as to minimize potential carry-over effects of emotional responses to script-driven imagery that may have influenced the FMD response.

#### Psychological and Demographic Assessment

Data collection began prior to the dissemination of Diagnostic and Statistical Manual– 5 [7]. Therefore, psychological assessments were based on the Diagnostic and Statistical Manual-IV [39]. PTSD symptom severity and diagnosis were assessed with the Clinician Administered PTSD Scale (CAPS—IV [58]). CAPS-IV interviews were conducted by a licensed clinical psychologist (RA), or a doctoral clinical psychology student (AC) under direct supervision. Given the variable time between CAPS and CV assessment (one week to six months), the primary outcome measure for the CAPS was PTSD symptom severity based on lifetime report. Full PTSD criteria was defined as a total severity score greater than or equal to 30 and full criteria for each symptom cluster (reporting frequency of at least “1” and intensity of at least “2” for at least 1 cluster B symptom, 3 cluster C symptoms, and 2 cluster D symptoms) on the CAPS-IV [58]. Similar to previously published methods [59], partial PTSD was defined as CAPS total severity score greater than or equal to 30, but not meeting full criteria for cluster C or D symptoms (missing 1 symptom).

Depressive symptoms were assessed with the Beck Depression Inventory II (BDI-II [60]). The Combat Experiences sub-scale of the Deployment Risk and Resilience Inventory (DRRI [61]), was used to assess level of combat experiences. Alcohol use was assessed with the Alcohol Use Disorders Identification Test (AUDIT [62]). Higher scores on self-reported measures of depression, combat, and alcohol use indicate increased depressive symptoms, increased number of combat experiences, and greater alcohol use, respectively. Height and weight were obtained to calculate body mass index (BMI). Participants were asked about prior history of traumatic brain injury (TBI) by asking them to report any experienced of “a concussion, blow to the head, or other head-related injuries”, the duration of loss of consciousness for the event(s), and any neurologic symptoms experienced with the event (i.e., dizziness, headaches, vision problems, etc.). Fourteen participants reported events consistent with mild TBI (LOC < 30 minutes), while individuals with history of moderate to severe TBI were excluded. Current smoking status was also collected. Participants were identified as non-smokers if they abstained from smoking for the past 12 months.

#### Physiological Assessment

Participants completed an anxiety-provoking task using script-driven imagery. This assessment is a slightly modified version of previously described script-driven imagery methods [49, 50]. Participants created four individualized scripts, including one each for negative, positive, neutral, and combat events. Participants were provided written instructions (modified from previous studies [49, 50] for script construction. Participants were instructed to write a description of each event (positive, neutral, negative, and combat), and include in that description bodily sensations they experienced at that time. Participants were also provided an example script, as well as a list of common physiological descriptors to aid in script generation. Scripts were recorded by a male research assistant and played back to the participant for two minutes, with a two-minute rest between scripts. The order in which participants heard negative, positive and neutral scripts was counterbalanced. The combat-related script was always played last to minimize prolonged carry over effects. Heart rate variability was obtained from 20 participants during script-driven imagery using an electrocardiogram (ECG) and corresponding software (CardioCard, Nasiff Associates, Central Square, NY). A frequency domain method (Fast Fourier Transformation) was used to analyze HRV [63]. Therefore, the LF/HF, which is thought to indicate balance between the sympathetic and parasympathetic systems, was used to index autonomic reactivity during script-driven imagery. The LF/HF was averaged for each script, with higher ratios indicating decreased parasympathetic activity. Data were extracted using QRSTool/CMetx software (Allen, Chambers, and Towers, Psychophysiology Lab, The University of Arizona), and processed using Kubios analysis software 2.0 (Biomedical Signal and Medical Imaging Analysis Group, University of Kuopio, Finland [64]). Data from three participants were excluded due to poor EKG data quality.

#### Endothelial Function

Flow-mediated dilation (FMD) was used to assess endothelial function, as an index of CV health. Participants were asked to refrain from food or caffeine for 12 hours and any medications prior to the procedure. Participants were asked to rest supine for 20 minutes prior to the start of the procedure in a temperature-controlled room (21–24 degrees C) that was quiet with dim lighting. We used a 9.0 Mhz linear array probe attached to a high-resolution ultrasound machine (Acuson, Sequoia 512, Siemens Medical Solutions USA, Inc., Mountain View, CA) to create an image of the brachial artery and blood flow. Our methods for the FMD procedure have been previously published [65–67].

Briefly, once the brachial artery was identified, a stereotactic clamp was used to stabilize the ultrasound transducer and hold it in place during the procedure. A resting baseline for diameter and blood flow velocity was recorded for 60-seconds. An automated blood pressure cuff (D.E. Hokanson, Bellevue, WA) was placed 2–3 cm below the anecubital fossa and was inflated above suprasystolic pressure to 220 mmHg for five minutes. Twenty seconds prior to cuff deflation, recording resumed for 3 minutes. All images were analyzed off-line using specialized software (Brachial Analyzer, Medical Imaging Applications, Coralville, Iowa).

### Statistical Analyses

Total severity scores calculated for the CAPS and the combat experiences sub-scale of the DRRI were the primary self-report variables of interest. We examined relationships with symptom severity rather than comparing dichotomous groups (i.e., PTSD versus non-PTSD) due to the fact that all participants had experienced trauma (combat exposure) and PTSD symptoms were normally distributed across the sample. Total severity scores were also calculated for the BDI-II and AUDIT. Autonomic reactivity was calculated by subtracting the LF/HF ratio during the neutral script from the LF/HF ratio for positive (POS-NEU), negative (NEG-NEU) or combat (COMB-NEU) scripts. LF/HF was also measured during baseline (2-minutes prior to script onset), and recovery (5-minute post combat script) periods. All LF/HF variables were log-transformed prior to analyses due to non-normal distribution. Relationships between these variables, as well as FMD, were then explored using two-tailed Pearson’s correlations. Exploratory correlations between FMD and known CV risk factors including age, current smoking status, and BMI were also conducted to determine possible covariates for the primary analyses.

The primary outcome measure for the dependent variable, FMD, was separated into a dichotomous measure of dilation less than or greater than five percent, with less than five percent indicating an impaired FMD response [33]. Variables in which the relationship with FMD was in the moderate to large effect size range were entered into a step-wise discriminant analysis to explore the predictive ability of FMD. Given the exploratory nature of the study, *p* < 0.05 was considered significant. Effect sizes are reported to aid in the interpretation of findings and inform the design of future studies.

## Results

Descriptive analyses of the current sample are presented in Table 1. Participants on average demonstrated impaired FMD (Table 1), with 15/24 participants exhibiting FMD < 5%. Age, mTBI history, alcohol use, BMI, and LF/HF during neutral scripts were unrelated to FMD and therefore not included as covariates (*p* > 0.10 for each variable). Excluding participants who were smokers did not alter study results.

**Table 1:** Descriptive statistics.

Variable	Mean	SD
Age	32.75	7.69
Education (years)	15.55	1.85
Smoking status	8.30%	-
mTBI	61.90%	-
FMD baseline diameter (mm)	4.39	.51
FMD % dilation	4.79	2.41
FMD dilation less than 5%	62.5%	-
CAPS total severity	57.50	32.56
BDI-II total	9.10	7.83
DRRI-combat	8.00	2.77
AUDIT	7.84	6.72
LF/HF Baseline	0.39	1.00
LF/HF Positive	0.82	0.82
LF/HF Neutral	0.35	0.65
LF/HF Negative	0.20	0.86
LF/HF Combat	0.45	0.85
LF/HF Recovery	0.76	0.85

Note: mTBI = mild traumatic brain injury; FMD = flow-mediated dilation; CAPS = Clinician Administered PTSD Scale; BDI-II = Beck Depression Inventory-II; DRRI-Combat = Deployment Risk and Resiliency Inventory—combat experiences sub-scale; AUDIT = Alcohol Use Disorders Identification Test; LF/HF = Fast Fourier Transformed low to high frequency power ratio of heart rate variability

**Fig 1:**
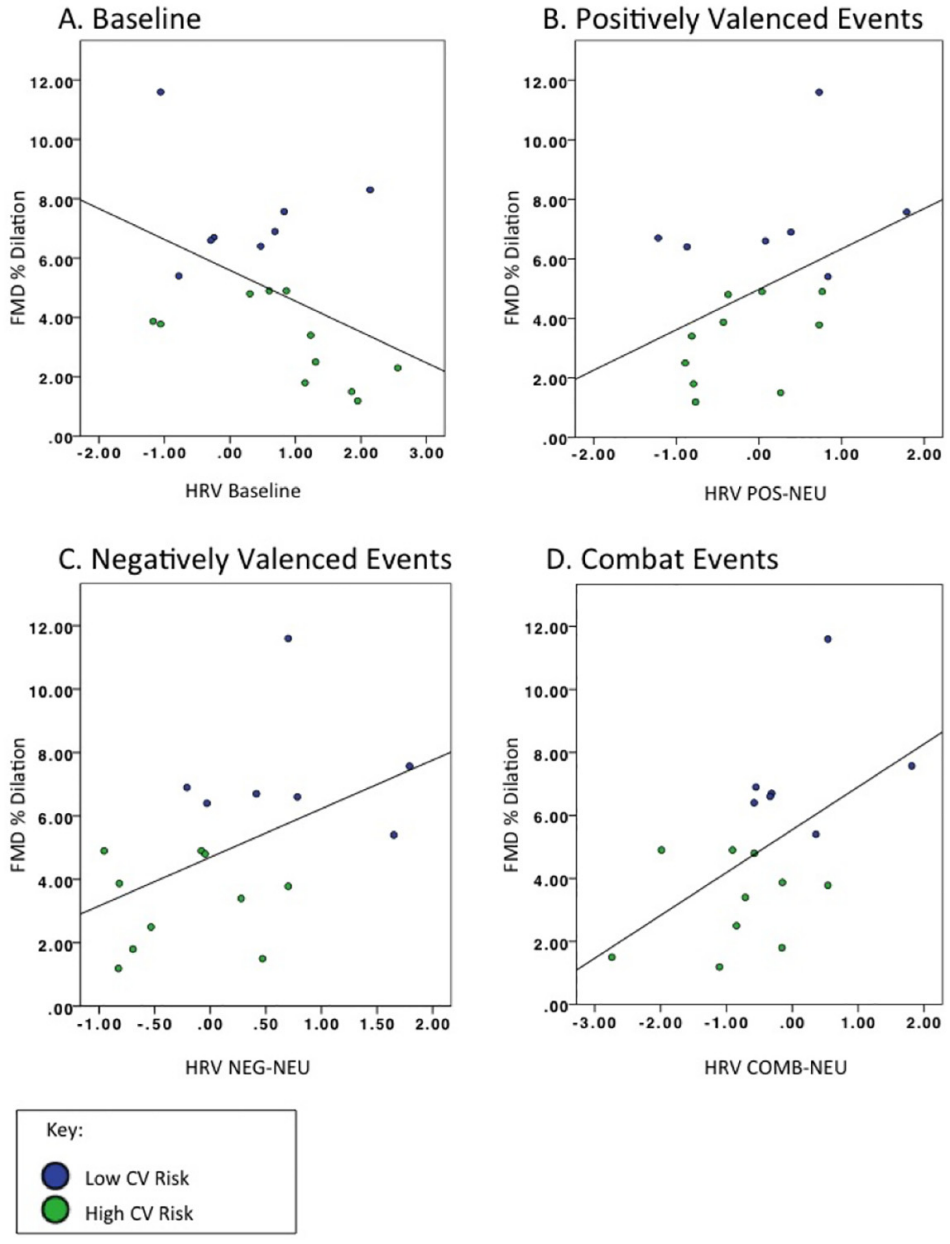
Relationships between autonomic reactivity to script-driven imagery and cardiovascular health. Relationships between autonomic reactivity (heart rate variability; low to high frequency power ratio) to script-driven imagery and flow-mediated dilation (FMD). Baseline = autonomic activity during baseline; POS-NEU = autonomic reactivity of positive scripts relative to neutral scripts; NEG-NEU = autonomic reactivity of negative scripts relative to neutral scripts; COMB-NEU = autonomic reactivity of combat scripts relative to neutral scripts; Recovery = autonomic reactivity during the recovery period; FMD = flow mediated dilation percent change; dilation less than 5% (green) indicates impaired FMD; dilation greater than 5% (blue) indicates FMD within the normal range.

PTSD symptoms related to combat experiences (R = -0.43, *p* = 0.037), but not to LF/HF ratio to emotional scripts (all *p*’s > 0.10). The relationship between PTSD and LF/HF at baseline was trending with a moderate effect size (R = 0.41, *p* = 0.082). Combat experiences were not significantly related to LF/HF at baseline or LF/HF reactivity to emotional scripts (all *p*’s > 0.10). Self-reported PTSD symptoms (R = -0.01, *p* = 0.968), depressive symptoms (R = 0.379, *p* = 0.068), combat exposure (R = 0.13, *p* = 0.534) and LF/HF during recovery periods (R = -0.35, *p* = 0.150) were not related to FMD. Greater LF/HF reactivity to positive scripts related to higher FMD (R = 0.526, *p* = 0.030) and there were trends for LF/HF reactivity to negative (R = 0.48, *p* = 0.052) and combat (R = 0.43, *p* = 0.085) scripts (Fig 1). A moderate to large effect size was found for higher LF/HF at baseline relating to lower FMD (R = -0.44, *p* = 0.058).

LF/HF at baseline, as well as LF/HF reactivity to positive, negative and combat scripts were entered into a stepwise discriminate analysis. LF/HF ratio to negative events emerged as the only significant predictor of impaired FMD (FMD < 5%; CC = 0.608, λ = 0.631, X^2^ = 6.685, *p* = 0.010), correctly classifying 76.5% of all cases.

## Discussion

Results from this pilot study provided partial support for our hypotheses. While psychological symptoms did not relate to FMD, we observed surprisingly low FMD values in our current sample of relatively young, physically healthy, combat veterans with only mild to moderate levels of PTSD. Secondly, results from discriminant analyses suggest that autonomic reactivity to emotional provocation, as measured by LF/HF, relates to poorer FMD in combat veterans and should be further investigated as a potential predictor or risk factor for poorer CV health. These pilot results have important implications for the screening and assessment of CV health in combat veterans and can be used to inform future research.

### CV Health in Combat Veterans

A large percentage (62.5%) of the current sample exhibited impaired FMD, with vessel dilation less than five percent (mean FMD = 4.79). In contrast, the average FMD reported for healthy adults is closer to seven and a half percent [33], which is significantly greater than observed in the current sample of combat veterans (Welsh’s t = 7.011, *p* < 0.001). This reduced FMD response was observed in our study population, despite their young age (M = 32.38), lack of significant alcohol and tobacco use, and moderate levels of PTSD symptoms. To our knowledge, two other studies have assessed the relationship between PTSD and FMD [39, 68]. Violanti and colleagues found that when compared to police officers with moderate levels of PTSD symptoms, those with high levels of PTSD symptoms showed significantly poorer FMD response [39]. This relationship was not altered by demographic or lifestyle factors. This previous study also reported relatively low FMD response [39], similar to what was observed in the current sample. A more recent study investigating PTSD and FMD in older adult veteran populations (mean age = 68 years) found that veterans with PTSD were more likely to exhibit poorer endothelial function relative to those without PTSD [68]. Importantly, Grenon and colleague’s study enrolled those with known CV disease, thus limiting the ability to determine if poorer endothelial function is a result of PTSD, age, or cardiovascular disease [68]. Taken together, these results suggest that impaired FMD response may be a by-product of high levels of trauma exposure or stress that can be exacerbated by severe levels of chronic PTSD. Exposure to traumatic and/or stressful events elicits an autonomic (fight or flight) response that can be adaptive in stressful situations [69]. It is therefore possible that even adaptive autonomic arousal subsequent to traumatic and/or stressful events may lead to endothelial dysfunction. Individuals who then go on to develop PTSD may therefore experience increased difficulty in regulating autonomic responses, which may then further exacerbate poorer CV health.

FMD provides the unique opportunity to assess endothelial-dependent function, adding to information provided by other measures of CV health (i.e., blood pressure, heart rate, lipid levels). The present results demonstrate the importance for physicians and healthcare providers to regularly assess for CV health with combat veterans (or other populations with high levels of trauma exposure), regardless of age or other demographic variables via measures of autonomic reactivity and/or FMD. Our findings highlight the importance of future research to identify factors that predict poor CV health in combat veterans and to identify targets for early intervention.

### Autonomic Reactivity and CV Health

Autonomic reactivity (HRV; LF/HF) to any emotionally valenced script (negative, positive, or combat-related) was found to positively relate to FMD (with a moderate to large effect size). This finding supports future investigations assessing the use of autonomic reactivity to emotional scripts as a predictor of CV health in populations with trauma history. Autonomic reactivity to negatively valenced stimuli emerged as the only significant predictor of impaired FMD. Interestingly, average LF/HF ratio to negative events was relatively lower than for neutral events suggesting decreased autonomic balance during neutral compared to negative events. This could reflect participant attempts to regulate autonomic arousal specifically during negatively valenced emotional provocation. It could be useful for future research to investigate whether one’s ability to purposefully down-regulate autonomic arousal may be an important contributor to chronic CV health, and a potential target for reducing impaired FMD.

The current pilot results also suggest that measures of autonomic reactivity may be more powerful predictors of CV health in combat veterans than self-report measures of psychological symptoms or combat experiences. This may be due to sympathetic and parasympathetic reactivity being a more direct indicator of adrenal reactivity and stress hormone release [70] than PTSD symptoms in general. In addition, measures of autonomic reactivity are less biased by retrospective recall, insight, clinical subjective judgments, or the under- or over-reporting of symptoms, than self-report measures or clinical interviews.

### Clinical Implications

The current findings suggest that autonomic reactivity to emotional provocation could potentially serve as a cost-efficient and objective screening measure for CV health. Moreover, this begs the question of whether interventions aimed at modifying autonomic reactivity could be beneficial in reducing impaired FMD. It is estimated that greater than 50% of CV disease reduction is attributed to changing CV risk factors [71]. Therefore, it is reasonable to speculate that if autonomic dysfunction is viewed as a CV risk factor, then interventions targeting autonomic reactivity could prove to be fruitful. Previous research provides strong evidence for biofeedback training, of either HRV or HR, for reducing CV risk [72]. Modulation of autonomic reactivity (specifically HRV) via regular exercise and stress management have reduced CV risk factors in patients with ischemic heart disease, above and beyond that of medication or treatment as usual [73]. More specifically, psychological research suggests that relaxation techniques such as progressive muscle relaxation are able to modulate physiological responses to visual stressors by reducing recovery time [74]. However, it would be important to test if 1) autonomic reactivity is a risk factor for the development of CV disease, and 2) interventions targeting autonomic reactivity decrease the propensity for developing CV disease in larger, longitudinal studies.

### Limitations

The current pilot study was limited by a small sample size (N = 24), lack of a non-trauma-exposed control group, and a cross-sectional design. The current sample included male veterans who have served in combat since the onset of OIF, limiting generalizability of findings to other types of trauma. Additionally, the symptom severity reported by veterans in the current study was in the mild to moderate range, which may have limited our ability to identify relationships between symptom severity, FMD, and autonomic reactivity. Future research should assess the potential impact of PTSD on autonomic reactivity and FMD in treatment-seeking veterans with more severe PTSD symptomology. Lastly, FMD is specific to endothelial-dependent dilation. It would be important for future studies to incorporate several modes of vasodilation (e.g. measures of endothelial and non-endothelial vasodilation) to further understand relationships to trauma, PTSD, and autonomic reactivity.

## Conclusion

Our results suggest that young combat veterans exhibit impaired FMD, and that autonomic reactivity to emotionally valenced events is a potential predictor of CV health. Previous research has established relationships between hyperarousal symptoms associated with PTSD and parasympathetic function [48–52, 75], as well as relations between depressed parasympathetic activity and CV disease [27, 76]. In contrast to previous research [22], but in line with research investigating the impact of combat exposure [10, 11], our results suggest that combat veterans in general (not just those with PTSD) may be at risk for poorer CV health. If replicated, the current findings emphasize the importance of regularly assessing CV health in all combat veterans, regardless of psychological diagnoses, and suggest that increased autonomic reactivity to emotional provocation is predictive of poorer CV health. These results provide initial support for future research investigating mediating relationships between trauma exposure, autonomic reactivity and CV health, as well as intervention research aimed at improving CV health.
